# Increased Risk of Autism Development in Children Whose Mothers Experienced Birth Complications or Received Labor and Delivery Drugs

**DOI:** 10.1177/1759091416659742

**Published:** 2016-08-09

**Authors:** Melissa Smallwood, Ashley Sareen, Emma Baker, Rachel Hannusch, Eddy Kwessi, Tyisha Williams

**Affiliations:** 1 Neuroscience Program, Trinity University, San Antonio, TX, USA; 2 Department of Mathematics, Trinity University, San Antonio, TX, USA; 3 Department of Biology, Trinity University, San Antonio, TX, USA

**Keywords:** autism, ASD, birth complications, induction, Pitocin

## Abstract

Autism spectrum disorder (ASD) is a perplexing and pervasive developmental disorder characterized by social difficulties, communicative deficits, and repetitive behavior. The increased rate of ASD diagnosis has raised questions concerning the genetic and environmental factors contributing to the development of this disorder; meanwhile, the cause of ASD remains unknown. This study surveyed mothers of ASD and non-ASD children to determine possible effects of labor and delivery (L&D) drugs on the development of ASD. The survey was administered to mothers; however, the results were analyzed by child, as the study focused on the development of autism. Furthermore, an independent ASD dataset from the Southwest Autism Research and Resource Center was analyzed and compared. Indeed, L&D drugs are associated with ASD (*p* = .039). Moreover, the Southwest Autism Research and Resource Center dataset shows that the labor induction drug, Pitocin, is significantly associated with ASD (*p* = .004). We also observed a synergistic effect between administrations of L&D drugs and experiencing a birth complication, in which both obstetrics factors occurring together increased the likelihood of the fetus developing ASD later in life (*p* = .0003). The present study shows the possible effects of L&D drugs, such as Pitocin labor-inducing and analgesic drugs, on children and ASD.

## Introduction

Autism spectrum disorder (ASD) is a pervasive developmental disorder ranging in severity, in which those diagnosed characteristically exhibit social difficulties, communication differences, and rigid or repetitive behavior (American Psychiatric Association, 2013; Gregory et al., 2013). The symptoms of ASD become apparent in early childhood, at around 2 to 3 years of age, and persist throughout an individual’s lifespan (American Psychiatric Association, 2013). The terms *ASD* and *autism* are used interchangeably in this article unless stated otherwise.

Recent statistics on the prevalence of ASD indicate a significantly increased rate of diagnosis over the past decade, with 1 in 68 American children being diagnosed with ASD in 2014 (Baio, 2014). While the increased diagnosis rate can be attributed in part to expansion of the diagnostic criteria, such as the inclusion of Asperger Syndrome in the Diagnostic and Statistical Manual of Mental Disorders IV, as well as increased awareness of ASD, increased exposure to environmental risk factors may also increase the prevalence of ASD in some populations. There has been ongoing research to ascertain the etiology of ASD; it is generally acknowledged that autism has a genetic basis, based on the evidence of a high level of heritability and high concordance rates in monozygotic versus dizygotic twins (Ronald et al., 2006; Hallmayer et al., 2011). However, this genetic basis is likely strongly influenced by environmental factors (Deth et al., 2008; Hallmayer et al., 2011).

This study examines one possible environmental risk factor: Exposure of the fetus to labor and delivery (L&D) drugs during labor and the birthing process. There has been a marked increase in the use of epidurals and labor-inducing drugs in the past 30 years, during which timeframe higher rates of autism diagnosis began occurring (Rice and Benson, 1961; Baio, 2014). Additionally, it has been reported that, although there is not sufficient evidence to claim independent risk factors, obstetric conditions were associated with an increased risk of autism, when factored along with parental age (Kolevzon et al., 2007). The two most commonly utilized classes of drugs in the L&D process are labor-inducing drugs and analgesic epidurals. The focus of this study analyzes uses of two classes of drugs that can be administered during the birthing process: induction and analgesic drugs. For example, Pitocin is a labor-inducing drug that has been in use since the late 1950s (Rice and Benson, 1961) and Bupivacaine is a local analgesic often used as an epidural, which is capable of crossing the placental barrier (Johnson et al., 1995; Capogna et al., 1999; Leighton and Halpern, 2002). There have been a few contradictory studies concerning the role L&D drugs might have in the development of autism. For example, data published within the past decade indicate the labor-inducing drug, Pitocin, may increase the risk of developing autism (Juul-Dam et al., 2001; Glasson et al., 2004; Wahl, 2004), while other recent publications contradict such findings and suggest there are no associations between the use of labor-inducing drugs and the risk of having a child diagnosed with autism at a later date (Gale et al., 2003; Newschaffer et al., 2007; Rodier et al., 2011). Consequently, further research is needed to understand the possible role of L&D drugs in the development of autism. Additionally, these previously published studies only focus on labor induction drugs and not analgesics, which are commonly administered (Gale et al., 2003; Wahl, 2004; Gregory et al., 2013; Gialloreti et al., 2014; Rosenstein et al., 2014; Weisman et al., 2015).

Therefore, using data collected from a survey conducted with mothers who have children with and without autism, we aimed to use this study to determine the effects of L&D drugs on the development of an autistic phenotype. The results of this study could have significance not only for the scientific community but also for the general population, namely expecting parents. This research has the potential to shed light on the possibility of environmental effects, specifically drugs administered during labor, on the development of autism. Greater understanding of the factors that may increase the risk of developing ASD could lead to changes in drug administration practices during the birthing process.

## Material and Methods

### 

#### Study population

The study was granted approval by the institutional review board at the Trinity University in San Antonio, TX. Participants provided written consent and responses to the survey were kept anonymous. Surveys were verbally administered through in-person or phone interviews.

Participants were comprised of mothers with children between the age of 2 and 25 who have been diagnosed with ASD. Mothers with age-matched neurotypical children served as controls. The participants completed a questionnaire aimed at collecting information regarding the labor experience for mother and children (Table 1). Although mothers served as participants to provide retrospective experiences, the study group of interest was the children. Therefore, the study included 49 children with an ASD diagnosis and 104 children who do not meet ASD diagnosis criteria.

**Table 1.** Labor and Delivery Risk Factors Assessed.

Factor	Code
Delivery style	1—Vaginal or 2—C-section
Location of birth	1—Hospital, 2—Home, 3—other-specified
Gestational age at labor	1—Full term (≥37 weeks) or 2—Pre-term (≤36 weeks)
Duration of labor	N/A—specific information recorded
Labor medications	Bupivacaine/Epidural, Pitocin, Both, None
Duration of labor medications	1—≤5.9 h or 2—≥6 h
Experienced birth complications	1—Yes or 2—No
Type of birth complication	N/A—specific information recorded

Additionally, a dataset of 101 ASD cases was obtained from the Southwest Autism Research and Resource Center (SARRC). This dataset was collected and analyzed independently, using our nonautistic sample as a control for comparison.

#### Data analysis

Questionnaire data were analyzed using Fisher’s test to determine significance between children with ASD and neurotypical children. Odds ratio was used as a post hoc analysis to determine the strength of significant results obtained via Fisher’s test. To determine if two independent factors could potentially have a confounding effect, the Cochran–Mantel–Haenszel (CMH) test was performed, followed by the Tarone post hoc test. All analyses were performed using the R (3.1.1) program with significance set to .05.

## Results

### Labor & Delivery Drugs and Autistic Children

**Figure 1.**
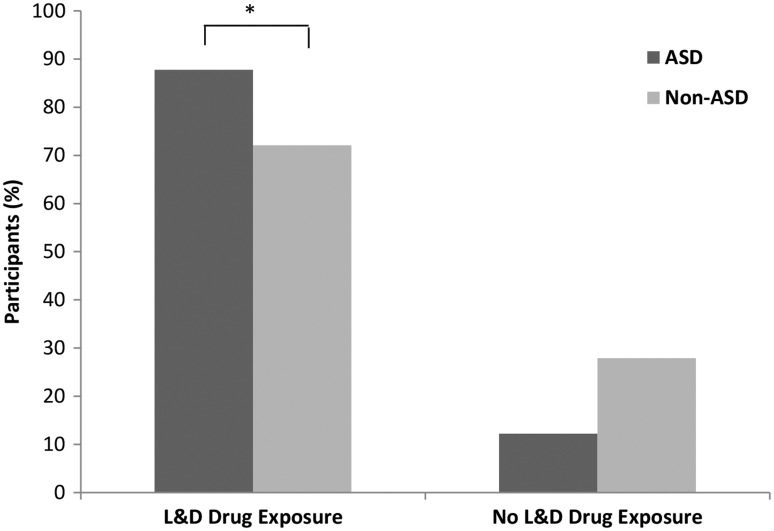
L&D drugs associated with higher rates of autism. ASD (*n* = 49) and non-ASD (*n* = 104) children were compared based on their exposure to labor and delivery drugs during childbirth. Children with ASD were 2.77 times more likely to have been exposed to L&D drugs during childbirth. *p = .039, OR = 2.77, Fisher’s exact test.

**Figure 2.**
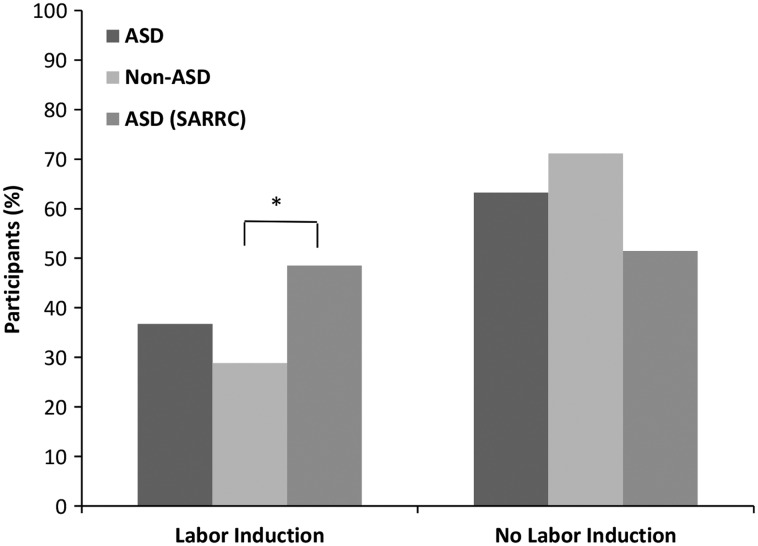
The labor-induction drug Pitocin was significantly associated with increased rates of Autism. ASD (*n* = 49) and non-ASD (*n* = 104) children were compared based on exposure to Pitocin during childbirth (*p* = 0.35). However, in an independent ASD (*n* = 101) dataset, children with autism were 2.32 times more likely to have been exposed to the labor induction drug than children without ASD (*n* = 104). *p = .004, OR = 2.32, Fisher’s exact test.

Based on the questionnaire completed by mothers of autistic and nonautistic children, it was found that 28% of children without autism and 12% of children with autism were not exposed to L&D drugs during childbirth, while 72% and 88% of each group, respectively, were exposed to an epidural, Pitocin, or a combination of the two during delivery (Figure 1). Moreover, it was found that individuals within the drug-exposed condition were 2.77 times more likely to exhibit an autistic phenotype than individuals who had not been exposed to L&D drugs during childbirth (*p* = .039). The data received from SARRC only included exposure to Pitocin. Therefore, to compare our findings to the SARRC data, we analyzed mothers who only received Pitocin during the child birthing process to mothers who did not receive any drugs. Within our dataset, labor induction was not significantly associated with ASD (*p* = .354). However, within the independent SARRC dataset, there was a significant relationship between these two factors (*p* = .004, OR = 2.32). Children with ASD in the SARRC sample were 2.32 times more likely to have undergone labor induction (Figure 2).

**Figure 3.**
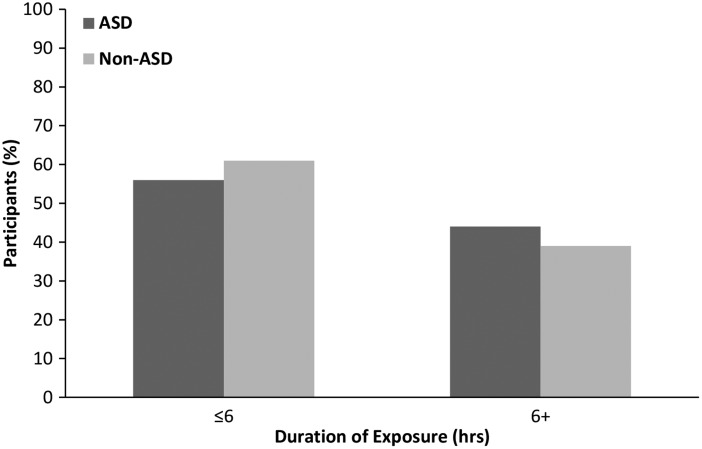
Duration of labor and delivery drug exposure is not found to be linked to autism. Children exposed to labor and delivery drugs for 6 h or greater were not more likely to be part of the ASD group (*n* = 41), as compared with the non-ASD (*n* = 70) children. *p* = .73, Fisher’s exact test.

Although our population of participants who were diagnosed with autism exhibited a higher proportion of L&D drug exposure than the control participants, there were still a sizable number of individuals in the exposed condition who remained neurotypical. This indicates that L&D drug exposure cannot be an independent factor accounting for the development of autism, suggesting that an additional factor(s) must be present or occur in order for the L&D drugs to have an impact on the development of an ASD phenotype. We sought to determine whether the duration of drug exposure during childbirth could play a role in risk of autism; however, our results showed duration of L&D drug exposure was not significantly associated with the development of ASD (*p* = .2; Figure 3).

**Figure 4.**
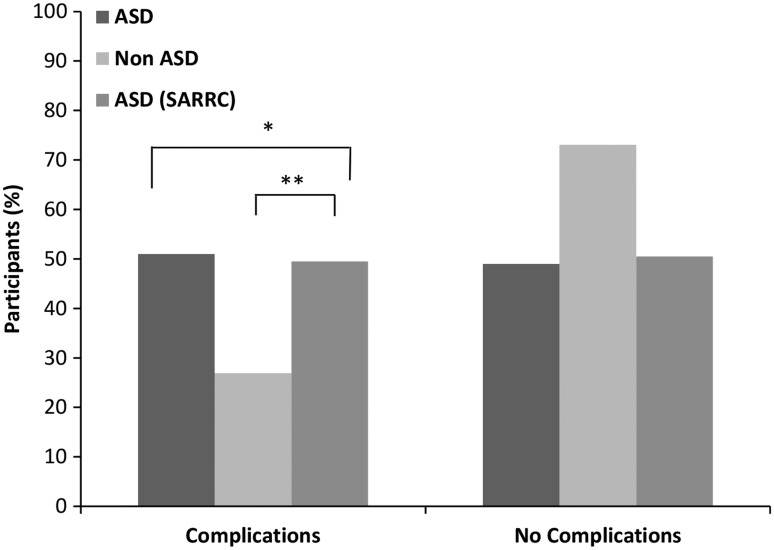
Mothers of children with ASD are more likely to experience birth complications. ASD (*n* = 49) and non-ASD (*n* = 104) children were compared based on the experience of a birth complication. Using the Fisher’s exact test, children with ASD were 2.83 times more likely to have been born after experiencing a birth complication, *p = .004, OR = 2.83. These findings were supported by the SARRC independent ASD (*n* = 101) dataset and non-ASD (*n* = 104). **p = .0009, OR = 2.66.

Within our dataset, birth complications were also found to be significantly associated with the development of ASD later in life. We observed that children with ASD were more likely to have experienced birth complications (*p* = .004, OR = 2.83). These findings were confirmed using the SARRC dataset (*p* < .001, OR = 2.66; Figure 4).

Since birth complications increase the likelihood of a mother receiving L&D drugs, we sought to determine whether the association between each of these factors was independent or if there was a confounding effect. Therefore, the CMH was performed, followed by the Tarone post hoc test. Our results from the CMH analysis revealed that there was no confounding effect, and birth complications and L&D drug exposure had a synergistic effect on the development of ASD (CMH = 12.97, *p* = .0003, OR = 2.3). The Tarone test supported the CMH data (*p* = .97). Additionally, as depicted in Table 2, the SARRC dataset verified our findings (CMH = 19.26, *p* = .00001, OR = 2.5).

**Table 2.** Labor and Delivery Drugs and Birth Complications Have a Synergistic Effect on Increasing the Likelihood of a Child Being Diagnosed With ASD.

	CMH statistic value	CMH *p* value	CMH pooled OR value	Tarone test *p* value
L&D drugs and birth complications vs. ASD	12.97	3.16 × 10^−4^	2.311	.97
Pitocin and birth complications vs. ASD	19.26	1.14 × 10^−5^	2.486	.75

CMH test and Tarone test results indicate that a combination of these factors can lead to higher rates of ASD than each factor independently. CMH = Cochran–Mantel–Haenszel; L&D = labor and delivery; ASD = autism spectrum disorder.

## Discussion

### A Mother’s Choice During Labor

It has been reported that the use of an epidural (either as an analgesic for labor pains or to perform a caesarean section) during the birthing process was more common to mothers of children with a diagnosis of ASD, as compared with controls (Glasson et al., 2004). Additionally, previous studies have suggested that epidurals can negatively impact the fetus, including impacting fetal core temperature and leading to low Apgar scores at birth (Glasson et al., 2004). Likewise, labor induction commonly occurs in obstetrics during the birthing process. While the role of oxytocin on pregnancy and labor has been well studied, the effects of administering exogenous oxytocin (Pitocin) during labor have not been well studied. Moreover, there have been conflicting reports regarding the role Pitocin might play in the development of ASD (Kenkel et al., 2014). Therefore, we analyzed the potential impact epidurals and Pitocin might have on the development of autism.

Results of our human survey study showed that children in the drug-exposed condition during labor were 2.77 times more likely to exhibit an autism phenotype. While the SARRC dataset only contained labor induction information, it was observed that mothers who received Pitocin during the birthing process were 2.32 times more likely to have a child diagnosed with autism later in life.

The fact that not all children exposed to an epidural (e.g. Bupivacaine), Pitocin, or both later developed an autism phenotype suggests interaction with other factors, which current research is attempting to elucidate. We found that the duration of the L&D drugs was not significantly associated with an ASD diagnosis later in life; however, experiencing a birth complication (e.g., fetal distress, pre-eclampsia, breech presentation, etc.) was significantly associated with ASD. These findings are supported by the independent dataset obtained from SARRC as well as a previous study, which shows children diagnosed with autism experienced more complications during birth, as compared with controls (Glasson et al., 2004). Furthermore, the L&D drug and the birth complications associations were found to have a synergistic impact as it relates the development of an ASD phenotype.

### Limitations and Future Directions

We conducted a study that explored the possible role L&D drugs might have in the increased prevalence of ASD, in attempts to better understand possible environmental factors and their connection to ASD. The results from our study demonstrate an association between the exposure to L&D drugs and the development of an autism phenotype, in support of previous reports with similar findings (Glasson et al., 2004; Wahl, 2004; Kenkel et al., 2014). However, it is very unlikely that L&D drugs alone would cause an autism phenotype; like any environmental factor, assessment of risk in the context of genetic predisposition should be performed.

Furthermore, we acknowledge that the current study has a few limitations. The first limitation is the sample size. We tried to address this limitation by including an independent dataset, the SARRC dataset. However, the SARRC dataset did not include all of the variables found in our survey (e.g., information about analgesic used during the birthing process). Furthermore, due to the sample size limitation, when the data were stratified for the type of L&D drug among the ASD group, the Pitocin only group comprised 6% of our dataset, while the Epidural only group comprised 48% and the Epidural + Pitocin group comprised 31%. This is in comparison to the SARRC dataset, where the Pitocin-exposed children comprised 64% of their dataset. Therefore, the divergent findings are likely due to the smaller sample size in our dataset. While this highlights the importance of a future independent study with a larger dataset to verify such associations found within the SAARC dataset, it does not reduce the significance of such findings. However, in the future, we would like to conduct a larger study to include collaborations with hospitals in order to recruit patients for prospective studies, which would also include data analyzed from medical records.

A second limitation is the fact that the data analyzed were questionnaire data, which can be subject to recall bias. An inherent known problem with association/case–control studies is recall bias. However, it has also been shown that the major problem with association studies that use parental reporting is not recall bias, but *nondifferential misclassification* (Infante-Rivard and Jacques, 2000). Two attempts were made to avoid this limitation: (a) careful selection of the questions included in the questionnaire (e.g., Yes or No questions or numerical ratings of 1 or 2) and (b) the included control group. Furthermore, while recall bias could possibly impact case–control studies, such studies have been extremely informative within the scientific community, despite some on the inherent limitations. Since the results from our human study demonstrate a relationship between the use of Pitocin and/or epidurals and the development of an autism phenotype, a future independent study with a larger sample size should be conducted.
